# Controlling Payload Heterogeneity in Lipid Nanoparticles for RNA-Based Therapeutics

**DOI:** 10.1101/2025.06.11.659145

**Published:** 2025-06-11

**Authors:** Turash Haque Pial, Sixuan Li, Jinghan Lin, Tza-Huei Wang, Hai-Quan Mao, Tine Curk

**Affiliations:** 1 Department of Materials Science and Engineering, Johns Hopkins University, Baltimore; 2 Department of Mechanical Engineering, Johns Hopkins University, Baltimore; 3 Institute for NanoBioTechnology, Johns Hopkins University, Baltimore; 4 Department of Biomedical Engineering, Johns Hopkins University School of Medicine, Baltimore; 5 Translational Tissue Engineering Center, Johns Hopkins University School of Medicine, Baltimore

**Keywords:** Lipid nanoparticles, RNA therapeutics, RNA encapsulation, kinetic Monte Carlo, molecular dynamics simulation, single-particle characterization

## Abstract

Lipid nanoparticles (LNPs), a leading non-viral nucleic acid delivery platform, are assembled with a heterogeneous payload distribution. This uneven therapeutic payload distribution can be critical parameters influencing delivery efficiency, therapeutic efficacy, and inflammatory side effects, limitations that become especially acute in prolonged gene therapies. Here, we integrate coarse-grained molecular dynamics and kinetic Monte Carlo simulations with single-particle characterization via cylindrical illumination confocal spectroscopy (CICS) and machine learning analysis to understand, step-by-step, the formation of RNA-loaded LNPs and the origins of the payload variability. We find that the balance between RNA diffusion kinetics and lipid self-assembly dynamics is the dominant driver of payload heterogeneity. Leveraging this mechanistic insight, we show that (i) finely controlled turbulent mixing minimizes payload variance and increases the uniformity of RNA distribution without altering LNP size, and (ii) systematic adjustment of salt concentration and PEG-lipid content tunes RNA loading in a volume-dependent manner. Together, these results elucidate the self-assembly landscape of LNPs and provide actionable design principles for crafting more uniform, potent, and safer LNP-based nucleic acid therapies.

## Introduction:

Nucleic acid-based therapeutics have revolutionized disease treatment, with lipid nanoparticles (LNPs) emerging as an effective delivery platform.^1–4^ The success of LNPs in FDA-approved vaccines, which deliver messenger RNA (mRNA), underscores their immense potential as a versatile delivery platform. Beyond vaccines, LNPs are being developed for various therapies, including small interfering RNA (siRNA)-mediated gene silencing,^5^ mRNA-based protein replacement,^6^ gene editing using mRNA and guide RNA,^7^ circular RNA for long-lasting protein expression in genetic disorder treatments,^8^ and DNA/RNA-based induction of immune responses against cancers and chronic infections.^9–11^ Despite this progress, a key gap lies in understanding and controlling the distribution of therapeutic payloads among LNPs, as heterogeneity in the payload distribution often leads to undesirable toxicity and reduced therapeutic efficacy.^12–14^ Therefore, establishing a comprehensive understanding and control of RNA-LNP formation both at the single-particle and population levels is needed for optimizing nucleic acid-based delivery systems, obtaining a desired immune response, and fine-tuning therapeutic outcomes.

RNA-LNPs are formed through a self-assembly process, where an aqueous solution of RNA is mixed with an alcoholic lipid solution containing ionizable lipids, PEGylated lipids, cholesterols, and helper lipids. Two key interactions primarily drive the self-assembly: the amphiphilic nature of the lipids, and electrostatic interactions between the negative RNAs and the positive ionizable lipids. However, this assembly process often produces LNPs with heterogeneous compositions, including LNPs without RNA (“empty LNPs”) and LNPs containing multiple RNA copies,^15^ characterized by a bimodal RNA loading distribution. This payload heterogeneity can significantly impact therapeutic outcomes, often leading to toxicity or reactogenicity.^12,13^ A recent study found that the higher mRNA loading reduces transfection potency, likely due to their deviation from the optimal lipid/RNA ratio.^16^ Additionally, studies have linked empty LNPs containing YSK13 lipids to liver toxicity, suggesting that the lipid component itself may contribute to adverse effects.^14^ Although less critical in vaccines, minimizing unwanted immune activation is critical in high-dose, long-term therapies such as gene delivery. Controlling the payload distribution is, therefore, essential to reduce toxicity, improve potency, enhance drug delivery consistency, and expand the applicability of LNP-based therapeutics.

Formulating LNPs with desired physical properties, such as average size and RNA encapsulation efficiency, is straightforward, especially with techniques like microfluidics.^17–19^ However, controlling and measuring payload heterogeneity remains a significant challenge. Recently, we developed Cylindrical Illumination Confocal Spectroscopy (CICS), enabling analysis of mRNA, siRNA, or other payload characteristics at the single-particle level.^15,20^ This method uncovered substantial payload heterogeneity and a bimodal loading distribution, with up to 50% of LNPs being empty. While bimodal distribution is typically caused by thermodynamic phase separation, it may also stem from kinetic factors. The key question is whether thermodynamic or kinetic effects dominate. If the cause is kinetic, enhancing mixing could reduce heterogeneity. On the other hand, if the cause is thermodynamic, allowing the LNP configurations to equilibrate via enhanced mixing would likely increase heterogeneity. Thereby, identifying the precise cause of heterogeneity is crucial for optimizing strategies to obtain the desired LNP payload distribution.

**Figure 1:**
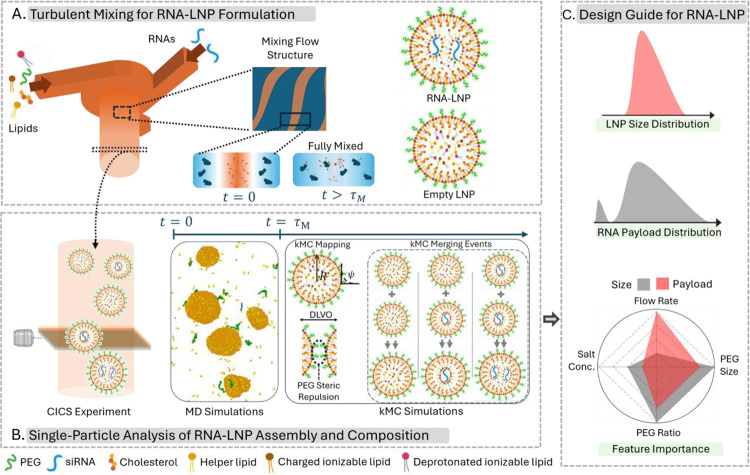
Overview of the RNA-LNP Self-Assembly Process: Experimental and Modeling Framework. (A) Schematic illustrating the self-assembly process of RNA-LNPs, where lipid-ethanol and RNA-aqueous solutions are mixed using a vortex mixer (left). Inlet flows generate turbulent shearing layers, keeping lipids and RNAs separate until full mixing occurs due to diffusion. This process resulted in RNA encapsulating and RNA-free LNPs (empty-LNP). (B) Cylindrical illumination confocal spectroscopy (left) is employed to analyze particle composition. Molecular dynamics (MD) simulations capture the initial stages of the assembly process (middle), whereas kinetic Monte Carlo (kMC) simulations are used for later stages (right). kMC incorporate charge regulation, Derjaguin-Landau-Verwey-Overbeek (DLVO) interactions, and PEG-PEG repulsion. Upon merging, LNPs update their composition and associated interactions. (C) kMC simulations provide detailed insights into RNA-LNP size distribution, payload distribution, and assembly dynamics. Coupling these simulations with machine learning enables the development of design rules for optimizing RNA-LNP formulations.

In this study, we systematically explore the origin of RNA payload heterogeneity and establish design principles to obtain a preferred LNP size and payload distribution. We also focus on the formation of empty LNPs and discuss how to minimize them. We first develop advanced multiscale computational techniques, employing molecular dynamics (MD) to model the initial stage of the RNA-LNP assembly process and kinetic Monte Carlo (kMC) to model the later stages, thus overcoming the challenge of limited simulation timescales. We investigate the effect of mixing rate, salt concentration ($c_{\text{salt}}$), and PEGylation on the LNP size and payload distribution; and use a random-forest machine-learning method to analyze the effect of these features. Findings are validated using the CICS single nanoparticle detection experiments (Fig. 1).

## Results:

We utilized a benchmark lipid formulation based on the FDA-approved siRNA drug Patisiran.^21^ Typically, RNA-LNPs are formed by mixing an RNA-containing aqueous solution with a lipid-containing alcohol solution in turbulent setups such as T-junctions,^22^ confined impinging jets,^23^ or multi-inlet vortex mixers.^24^ We used a 3-inlet vortex mixer at total flow rates of 10, 20, and 30 mL/min, with a siRNA-to-lipid flow rate ratio of 3:1. After mixing and subsequent dialysis (conducted 1-hour post-mixing), the siRNA-LNPs were analyzed using CICS (at 18 hours post-mixing), where each particle passes through a detector individually. Fluorescence signals from the detector enabled differentiation among LNP populations, categorizing them into siRNA-encapsulated LNPs, empty LNPs, and free unencapsulated siRNAs (Figure 1A–1B). This experimental system served as a reference for parameter selection and validation in our modeling approach.

### MD simulations reveal the origin of empty LNPs

**Figure 2:**
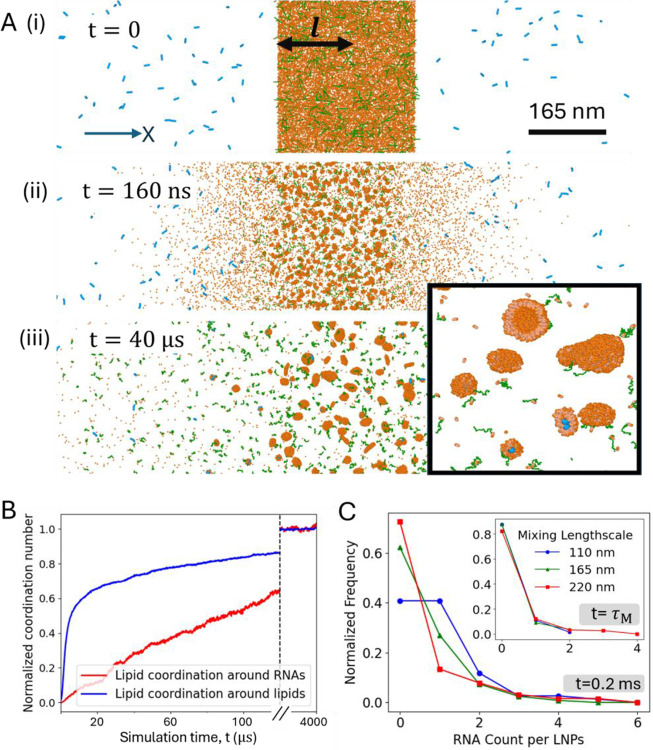
Molecular Dynamics (MD) Simulation of siRNA-LNP Assembly. (A) CG-MD simulation depicting the initial distribution of siRNA (blue), lipids (orange), and PEG (green) at (i) t = 0, (ii) t=1600τ, and (iii) t=40000τ(τ=2ns). The characteristic mixing length scale, l(165 nm for these snapshots), is shown in black. Over time, lipids begin to aggregate and form small nanoparticles. Inset shows a zoom-in of the simulation, showing empty and siRNA containing LNPs. (B) Number of RNA-lipid, and lipid-lipid contacts: normalized with the coordination number of RNA and lipid in the first solvation shell, respectively. (C) RNA payload distribution for different mixing length, l, at $t=10^{5}\;\tau$ (or 0.2 ms). RNA distribution at mixing time scale $\tau_{M}=l^{2}/2D$, collapses to a master curve (inset). The $\tau_{M}$ values are about 25μs,57μs, and 100μs for l=110,165, and 220 nm, respectively.

To investigate the initial stages of the siRNA-LNP self-assembly process, we employed coarse-grained (CG) MD simulations. In turbulent mixing setups, the kinetic energy from the inlet flows breaks the flow jets into inter-shearing layers or turbulent eddies as the solutions enter the mixing chamber.^25^ To replicate these inter-shearing layers in simulations, we initially divided the system into distinct siRNA and lipid layers along the X direction, maintaining a volume ratio 3:1 (Figure 2A(i)). Simulations reveal that during the mixing process, most lipids quickly aggregate to form small nanoparticles before significantly interacting with siRNA (Figure 2A, 2B). This early lipid aggregation leads to the formation of primarily empty LNPs. To our knowledge, this represents the first MD simulation that captures the RNA-LNP mixing process in a manner consistent with experimental conditions.

The thickness of inter-shearing layers can be controlled by adjusting the flow rate during siRNA-LNP formation.^25^ At higher flow rates, the increased kinetic energy leads to more intense breakdown of the flow and smaller turbulent eddies.^26^ In contrast, smaller flow rates may result in laminar flow, without any turbulent eddies. Following Ref. 25, we define the characteristic mixing length scale, l, as half the lipid layer thickness (Figure 2A). The distribution of siRNA on LNPs was analyzed at a simulation time of 100,000τ (simulation time unit τ=2ns, yielding a total assembly time of 0.2 ms) for varying l. The results show that thicker inter-shearing layers (correspond to slower mixing rates in experiments) lead to a higher proportion of empty LNPs compared to thinner layers (Figure 2C). To quantitatively explain this behavior, we calculate the time required, $\tau_{\text{M}}$, for siRNA and lipids to diffuse across the mixing length scale, $\tau_{\text{M}}=\frac{l^{2}}{2D}$ where D is the inter-diffusion constant (noting that in these simulations, $\tau_{\text{M}}$ values are about 25μs,57μs, and 100μs, respectively). Since individual siRNA molecules are much smaller than the LNPs (Figure 2A), we approximate $D\approx D_{\text{siRNA}}$. The estimation of $D_{\text{siRna}}$ is discussed in the SI. We find that at $\tau_{\text{M}}$ the loading distribution for different l collapse into a master curve (Figure 2C, inset). This indicates that the distribution of siRNA on LNPs is primarily governed by the diffusion timescale $\tau_{\text{M}}$, which is influenced by the thickness of the inter-shearing layers.

### kMC simulations and CICS experiments show the effect of mixing flow rates

**Figure 3:**
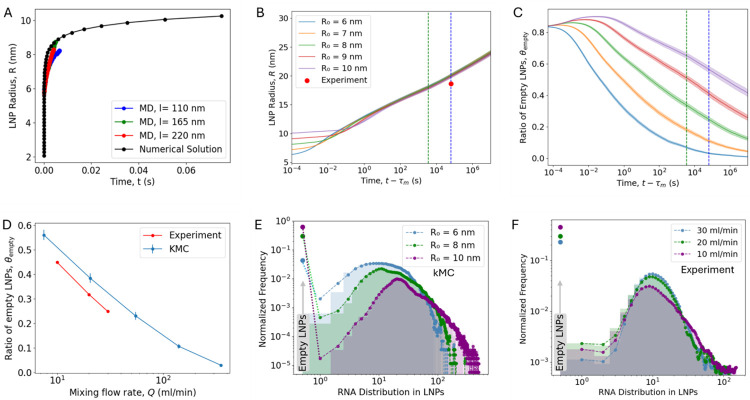
Effect of Mixing Flow Rate in LNP Size and Payload Distribution. (A) Mean-field theory prediction for LNP growth and coarse-grained MD simulation results. This provides the initial LNP radius input for the kMC simulations. (B) Time evolution of the average LNP radius from kMC simulations. (C) Time evolution of the ratio of empty LNPs, defined as the fraction of LNPs without siRNA. The green vertical lines denote t = 1 h (post-mixing dialysis), and the blue lines denote t = 18 h (to match experimental measurement via CICS). (D) Ratio of empty LNPs as a function of mixing flow rate Q at t=18 h. siRNA loading distribution at t=18 hours from kMC (E) and experiment (F). In both (E) and (F), normalized frequency distributions show a bimodal profile, with empty LNPs indicated on the left. Parameters: PEG MW is fixed at 2000 Da $\left(R_{F}=3.6\;nm\right)$, PEG ratio 1.5% of total lipids, and the initial salt concentration $c_{\text{salt}}=0.025\;M$. Dashed line are smoothed to guide eyes.

Since MD simulations cannot capture the full growth dynamics of LNPs due to time scale limitations, we employ kMC simulations to model the aggregation of LNPs on timescales larger than the mixing time $\tau_{\text{M}}$. kMC simulations enable dynamic evolution of LNP growth^27^ and allow for the investigation of payload and size distributions. To initialize kMC simulations, we estimated the LNP radius at $\tau_{\text{M}}$, denoted $R_{0}$, for kMC (at $\tau_{\text{M}}$), using a mean-field coalescence equation to model the temporal growth considering no effect from RNAs (Figure 3A; see Supplementary Information for derivation). We validated this approach by comparing the predicted sizes to those obtained from MD simulations. While theoretical models assume spherical nanoparticles, MD simulations revealed a distribution of morphologies, including spherical and disk-like structures. To facilitate consistent comparison with the mean-field model, we computed the mass of each LNP in the MD trajectory and converted it to an equivalent spherical radius. This yielded strong agreement between MD and theory, though the slightly slower growth observed in MD can be attributed to the reduced diffusion rates of non-spherical particles compared to spheres. Based on MD simulation results (Figure 2C, inset), we set that 83% of the LNPs are empty at $\tau_{\text{M}}$. In the turbulent mixing regime (flow rate Q≥10ml/min), the characteristic mixing time depends on the flow rate as $\tau_{\text{M}}=b1/1.75\times Q^{-\alpha 1}$, where $b1=1.3\times 10^{3}$ and α1=1.5,^25,28^ which allows us to compare experimental observations with kMC simulations. A faster flow rate results in more turbulent mixing conditions with smaller l and shorter $\tau_{\text{M}}$ (Figure 2), and thus smaller size $R_{0}$ (Figure 3A).

Figure 3B illustrates the evolution of the average LNP radius R (note R is the bare LNP radius that does not include the PEG chains) in the kMC simulation. Surprisingly, particles reach an average radius of approximately 20 nm at 18 hours regardless of the initial size $R_{0}$. This similarity in particle size for different $R_{0}$ is also observed in experiment. Dynamic light scattering (DLS) data reveals weak dependance of particle size on the mixing rate, with radii of 50.3 nm, 51.9 nm, and 52.5 nm at flow rates of Q=10ml/min,Q=20ml/min, and Q=30ml/min, respectively. This weak correlation is consistent with previous studies, which demonstrated that nanoparticle size decreases with increasing flow rates in laminar mixing regime but plateaus in the turbulent flow regime.^25,29^ Finally, according to prior LNP experiments, DLS-derived average radii are about 2.8 times larger than those measured by cryo-TEM^15^ because DLS is skewed towards large particles, and we find that rescaling the DLS data agrees very well with the kMC simulations (average of LNP size from experiment is shown in Figure 3B).

To investigate the effects on LNP loading distribution, we analyzed the time-dependent changes in the ratio of empty LNPs $\theta_{\text{empty}}$ (Figure 3C), which is defined as the fraction of LNPs that contain no RNA, $\theta_{\text{empty}}=N_{\text{empty}}/N_{\text{total}}$, with $N_{\text{total}}$ the total number of LNPs and $N_{\text{empty}}$ the number of LNPs containing no RNA. As LNPs merged into larger particles, the ratio of empty LNPs decreased overall. However, unexpectedly, the ratio increased in the early stages, which can be explained by the stronger electrostatic repulsion between empty LNPs, leading to LNPs containing siRNA to merge more quickly. Figure 3C demonstrates that reducing the initial LNP size significantly reduces the ratio of empty LNPs. Quantitative comparison between kMC results and experimental data at different mixing flow rates shows excellent agreement (Figure 3D). While previous studies have shown the controllability of average nanoparticle size and drug loading efficiency, the distribution of internal components has remained largely unexplored. Our findings show that rapid mixing improves the homogeneity of the internal composition within the nanoparticles. Surprisingly, this implies that the bimodal loading distribution results from mixing kinetics rather than thermodynamic phase separation. This is further supported by analyzing the entire siRNA loading distribution within LNPs (kMC data in Figure 3E, CICS data in Figure 3F). A larger initial LNP radius (smaller flow rate) results not only in more empty LNPs but also increases the number of LNPs with excessive siRNA (longer distribution tail), indicating highly heterogeneous encapsulation and a broader bimodal distribution. The agreement between kMC and experimental data (Figure 3B, D-F) is remarkable given that the kMC model contains no fitting parameters. This strongly suggests that the kMC model captures the relevant physical processes and can be used as an independent tool to predict LNP assembly.

### kMC simulations show the effect of PEG size and salt concentration

Along with mixing setup, PEG molecular weight (MW) is crucial in LNP assembly, stability, and delivery efficiency.^30,31^ PEG forms a hydration shell that prevents LNP aggregation and extends circulation time. LNPs with shorter PEG (e.g., 750 Da) show little difference from PEG-free LNPs. However, longer PEG chains (over 5000 Da) can hinder cellular uptake and endosomal escape, key steps in delivering therapeutic agents. Therefore, understanding the intermediate range (1000–5000 Da) is critical for optimizing stability and RNA delivery.

**Figure 4:**
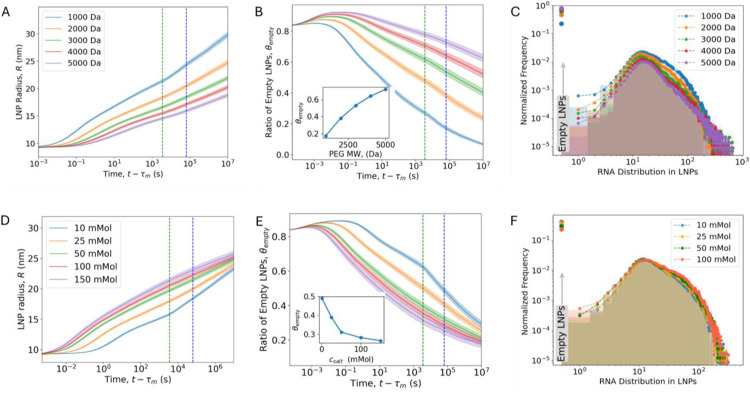
Effect of PEG size and salt concentration on LNP size and payload distribution. (A) LNP growth and (B) ratio of empty LNPs from KMC simulations for varying PEG MW. The green vertical line denotes t = 1 h (post-mixing dialysis), and the blue line denotes t = 18 h (to match experimental measurement via CICS). Inset in (B) shows the ratio of empty LNPs for varying PEG MW at 18 hours. (C) siRNA loading distribution at t=18 hours from kMC. Parameters: Initial LNP radius is fixed at 9 nm, PEG ratio 1.5% of total lipids, and the initial salt concentration $c_{\text{salt}}=0.025\;M$. (D) LNP growth and (E) ratio of empty LNPs from kMC simulations for different $c_{\text{salt}}$. Inset in (E) shows the ratio of empty LNPs for varying salt concentration at 18 h. Parameters: Initial LNP size is fixed at 9 nm, PEG ratio is 1.5% of total lipids and PEG MW is 2000Da $\left(R_{F}=3.6\;nm\right)$.

We investigated the effect of PEG MW by adjusting the Flory radius, $R_{\text{F}}=\text{a}n^{3/5}$ in PEG-PEG steric repulsion calculation (a=0.37 nm is the monomer size and n the degree of polymerization).^32,33^ Figure 4A shows that increasing the PEG MW slows LNP growth, creating an inverse relationship between PEG MW and LNP size. This well-known effect^31^ is explained by the increased steric barrier $E_{\text{PEG}}$ resulting from a larger $R_{\text{F}}$ (see Eq. 2 in the Methods section). Conversely, we find that the ratio of empty LNPs increases monotonically with increasing PEG MW (Figure 4B); with increasing PEG MW, LNP growth kinetics become slower, resulting in smaller and a higher fraction of empty LNPs. As $\theta_{\text{empty}}$ increases with PEG MW, we observe a reduction in the normalized frequency of RNA loading. While no clear peak shift is observed, a subtle broadening of the distribution tail is evident for the highest PEG MW (Figure 3C). This behavior reflects an increase in the variance of the payload distribution, where the modal frequency decreases and the tail extends. A similar effect is expected when increasing the PEG fraction, as both variables influence the PEG-PEG repulsions (see Eq. 2 and ref. 15).

In addition to PEG, salt concentration $c_{\text{salt}}$ plays a key role in the self-assembly of LNPs by modulating electrostatic interactions. We explore an experimentally relevant range of $c_{\text{salt}}$ from 10 mM to 150 mM. kMC simulations reveal that increased $c_{\text{salt}}$ accelerates aggregation by reducing electrostatic repulsion between cationic LNPs, as shown in Figure 4D. Beyond 50 mM, $c_{\text{salt}}$ yielded similar aggregation kinetics, due to neutralization of surface charge and vanishing electrostatic surface potential. Additionally, the faster aggregation at higher $c_{\text{salt}}$ reduces the proportion of empty LNPs (Figure 4E and inset). Changes in $c_{\text{salt}}$ yield a similar effect on siRNA distribution (Figure 4F) as changes in the PEG MW (Figure 4C), although the effect of salt is less pronounced compared to PEG.

### Analysis of LNP size and payload polydispersity

**Figure 5:**
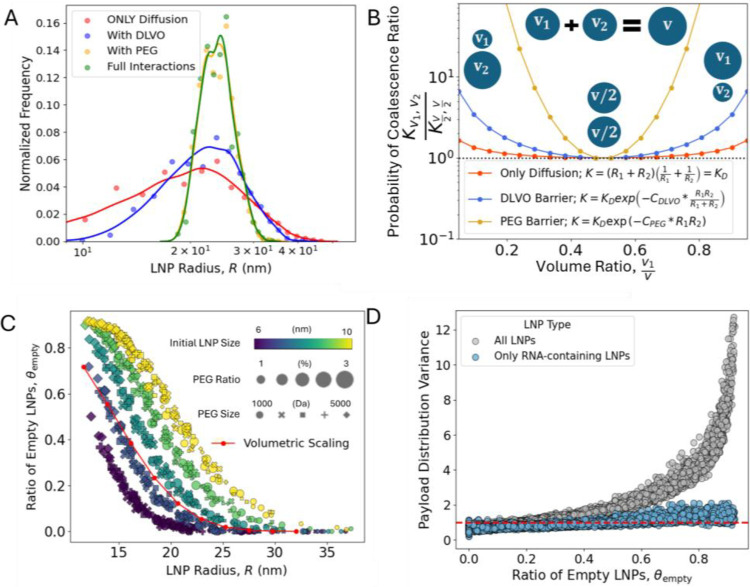
Analysis of Size and Payload Distribution Polydispersity. (A) LNP size distributions from kMC simulations using different aggregation kernels. Kernel density estimation (KDE) lines are overlaid to highlight the distribution profiles. All simulations start with N = 2000 LNPs and proceed until only 100 LNPs remain. (B) Aggregation bias expressed as the ratio $K_{v_{1},v_{2}}/K_{v_{/2},v/2}$, i.e., the probability of coalescence between an LNP of volume $v_{1}$ and one of volume $v_{2}$, relative to the probability of coalescing two LNPs of volume v/2 to form a larger LNP of volume v. This illustrates size-selective merging behavior. (C) Correlation between the ratio of empty LNPs and the average LNP radius at t = 18 h from kMC simulations. The red line indicates a volumetric scaling, with $R_{0}=10$ and $\theta_{0}=0.83$. (D) Correlation between the ratio of empty LNPs and the pooled payload distribution variance across LNP populations.

The above findings, combining kMC simulations and experimental data, highlight the complex interplay between mixing behavior, steric repulsion, and electrostatic interactions in LNP coalescence and growth. To further unravel precisely what factors determine the LNP heterogeneity; we isolate and analyze how different parameters affect LNP size and payload distribution by selectively deactivating the contributions from PEG (Eq. 2) and DLVO interactions (Eq. 3). Excluding both PEG and DLVO interactions results in LNP coalescence driven solely by diffusion, leading to a very broad LNP size distribution (Figure 5A). Minimizing size polydispersity is crucial for improving the performance, stability, and safety of LNPs.^1^ Reintroducing either DLVO or PEG interactions narrows the distribution, with PEG having a significantly stronger effect.

To understand why PEG exerts greater influence, we examined the aggregation kernel K and the associated fusion energy barriers to understand size-selective merging behavior. For PEG-PEG repulsion, the fusion barrier $E_{\text{PEG}}$ between two spheres with radii $R_{1}$ and $R_{2}$ is $E_{\text{PEG}}=C_{\text{PEG}}R_{1}R_{2}$ and for DLVO interactions it is $W=C_{\text{DLVO}}\frac{R_{1}R_{2}}{R_{1}+R_{2}}$ (Eqs. 2 and 3). Here, $C_{\text{PEG}}$ and $C_{\text{DLVO}}$ are simplified multiplicative factors, derived using typical values in Eqs. 2 and 3. In Figure 5B, we plot the aggregation bias ratio $\frac{K_{v_{1},v_{2}}}{K_{v/2,v/2}}$, representing the probability of coalescence between an LNP of volume $v_{1}$ and with another LNP of volume $v_{2}$, relative to the probability of merging two LNPs of volume v/2 to form a larger LNP of volume v. This ratio reveals that the probability of merging a smaller LNP with a larger one is higher than the probability of merging two LNPs of similar sizes. This behavior promotes a narrower size distribution by: (1) reducing the number of smaller LNPs and (2) limiting the formation of very large LNPs. We find that this effect is stronger for PEG compared to DLVO, as the DLVO fusion barrier increases exponentially with LNP radius, whereas the PEG fusion barrier increases as a squared exponential. Thus, PEG steric repulsion is the main factor that determines the LNP size and size polydispersity, with DLVO electrostatic repulsion having a smaller effect.

Having analyzed the size polydispersity, we now turn to payload distribution. Investigating the correlation between LNP size and the payload distribution, and whether these properties can be independently controlled, is crucial for optimizing LNP formulations. We find that the ratio of empty LNPs, $\theta_{\text{empty}}$, and the average LNP radius R behave in a manner close to volumetric scaling: $\theta_{\text{empty}}=\theta_{0}^{m}$, where $\theta_{\text{empty}}$ is the fraction of empty LNPs at radius $R,\theta_{0}$ is the fraction of empty LNPs at $R_{0}$, and $m={\left(R_{m}/R_{0}\right)}^{3}$ is the average number of merging events to grow an average particle size from $R_{0}$ to $R_{m}$. The slight deviation of kMC results compared to volumetric scaling (Figure 5C) is attributed to the non-constant aggregation kernel, as the probability of merging is not equal for all LNPs. Thereby, mechanistically, changes in PEG or salt concentration directly affect the LNP size and size polydispersity, while their effect on the payload distribution is largely indirect; a secondary effect due to the change is size.

We further investigated the relationship between overall payload heterogeneity and the fraction of empty LNPs by analyzing the pooled variance of siRNA loading distributions across LNP populations (Figure 5D). Two metrics were computed: (1) the total variance across all LNPs, including empty particles, and (2) the variance restricted to RNA-containing LNPs only. In both cases, we observed that the variance in payload distribution increases monotonically with the fraction of empty LNPs. This indicates that heterogeneity is amplified when a substantial portion of the population is in empty LNPs. Notably, even when empty LNPs are excluded from the analysis, the intrinsic variance among RNA-loaded particles still increases with $\theta_{\text{empty}}$. This suggests that inefficient mixing or unfavorable assembly conditions not only result in more empty particles but also lead to greater variability in payload among loaded LNPs. For instance, this effect is clearly illustrated in Figure 4C for high PEG molecular weight conditions, where the modal frequency decreases and the distribution tail broadens, indicating higher payload variance. Together, the results in Figure 5 establish a foundational understanding of the underlying correlations, offering a framework for decoupling and independently tuning LNP size and RNA loading distributions.

### Machine learning elucidated design rules

**Figure 6.**
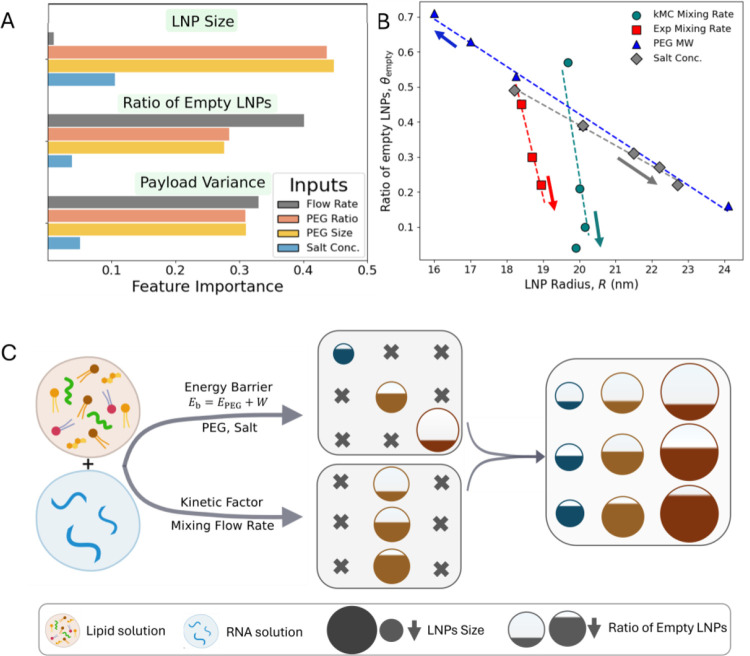
Design Guide for LNP Size and Payload Distribution. (A) Random-forest feature importance analysis highlights the relative importance of different parameters in controlling LNP size and payload. (B) Mixing rate affects the ratio of empty LNPs $\theta_{\text{empty}}$, but hardly modify the LNP size R, in both kMC and experiment. Volumetric trend is observable for PEG and salt concentration. (C) Schematic shows the kinetic and non-kinetic factors influencing LNP size and payload distribution. Energy barrier in merging can control LNP size and payload, volumetrically. For a particular size, payload can be controlled by kinetic measure (e.g. mixing flow rate). By integrating both kinetic and non-kinetic factors, a wide range of LNP sizes and payload distributions can be achieved.

To identify and quantify the most influential factors affecting LNP size and payload distribution, we performed a feature importance analysis using a random forest-based ensemble machine learning approach^34^. Feature importance quantifies how much each input variable (feature) contributes to output predictions. Our input parameters space includes: $R_{0}$ (6 to 10 nm), PEG MW (1000 to 5000 Da), PEG ratio of lipids (1 to 3%), and ${\text{c}}_{\text{salt}}$ (10 to 150 mM). The analysis reveals that the initial LNP size or mixing flow rate has a negligible effect on the final average LNP size (Figure 6A), whereas the PEG molecular weight (MW) and the PEG ratio play the most significant roles in determining LNP size. ${\text{c}}_{\text{salt}}$ can also influence LNP size, but its effect is constrained by the upper limit (charge neutralization for ${\text{c}}_{\text{salt}}\;>\;50\;\text{mM}$), as well as a relatively weaker effect compared to PEG (as seen in Figure 5B). Interestingly, the flow rate emerges as the most critical factor controlling the ratio of empty LNPs and the payload distribution, despite having minimal influence on the final LNP size (Figure 6A).

The LNP size and empty LNP ratio data from Figures 3 and 4 are summarized in Figure 6B. Changes in mixing rate yield a nearly vertical slope on the $\theta_{\text{empty}}-R$ parametric plot, observed in both computational and experimental data, highlighting the independence of controlling payload distribution from LNP size. In contrast, the gradual slopes for PEG and salt indicate a strong correlation between LNP size and payload distribution, showing a volumetric trend.

## Discussion:

Kinetic Monte Carlo and experimental results demonstrate that the size and payload distribution of LNP can be controlled independently (Figure 6A, B). These insights can be used to produce LNPs with controlled size and payload distribution (Figure 6C). The molecular weight of PEG, the PEG ratio, and salt concentration all contribute to the LNP merging energy barrier (Figure 5), which determines the final size of the LNPs. Thus, by adjusting the PEG MW, the PEG ratio, or the salt concentration, we can precisely control the average size of the LNPs. In contrast, the mixing flow rate acts as a kinetic factor, influencing the relative assembly of lipid-lipid and RNA-lipid, thereby affecting the distribution of the payload. RNA payload distribution can be fine-tuned independently of the LNP size through careful control of the mixing process. This level of precision is crucial, as LNP size significantly impacts therapeutic effectiveness by affecting cellular uptake, while homogeneity is important for ensuring both potency and safety.

Our computational framework is generalizable to a broad class of self-assembly systems. Within the kMC model, the chemical characteristics of lipid components can be systematically tuned by adjusting parameters such as charge regulation and molecular mass. Moreover, key experimental variables, including the duration of dialysis, total assembly time, and the ratio or amount of different molecular components, are explicitly controllable in the simulation. The framework is also adaptable to diverse payloads, provided their mass and charge properties are properly defined. Overall, our simulation results show good agreement with experimental observations, supporting the validity of the approach. Despite its broad applicability, opportunities remain for refinement. For instance, incorporating proper interdiffusion kinetics in MD simulations could enhance accuracy when modeling large payloads comparable in size to the LNPs themselves. Additionally, our current feature importance analysis is limited to the parameter space defined by the training data and does not yet account for other important controlling factors such as flow mixing ratios (FRR) or nitrogen-to-phosphate (N/P) ratios, which may further influence LNP formation and heterogeneity.

In conclusion, through the integration of CG-MD and kMC simulations, and CICS single-particle spectroscopy, we have developed a comprehensive understanding of the factors influencing LNP formation and siRNA payload distribution. Our simulation shows that the origin of empty LNPs and bimodal payload distribution is kinetic rather than thermodynamic; rapid mixing significantly reduces empty LNPs and narrows the siRNA payload distribution without altering LNP size. While PEGylation and salt concentration also affect payload distribution, they do so only indirectly by changing the average LNP size. Additionally, kMC data shows that controlling the fusion barrier through PEG modification, rather than electrostatic stabilization, is crucial for achieving narrow size and payload distributions. These findings offer essential design principles for optimizing LNP-based RNA therapeutics, aiming to enhance both their efficacy and safety. Additionally, the open-source computational framework we developed is freely accessible and can be extended to other therapeutic components, such as mRNA, and other nanoparticles, offering a powerful tool for efficiently screening nanoparticle formulation parameters.

## Methods:

### Single particle analysis of siRNA LNP on CICS

For the single nanoparticle analysis of siRNA LNP, the LNPs use the same formulation stoichiometry as used in simulations, with DLin-MC3-DMA (MedKoo Biosciences, Cat.#555308), DSPC (Avanti Polar Lipids, Cat.#850365), cholesterol (Sigma-Aldrich, Cat. #C8667), DMG-PEG2000 (NOF America, Cat# GM020) at a molar ratio of 50:10:38.5:1.5 dissolved in 100% ethanol and luciferase mRNA in 25 mM sodium acetate buffer at pH 4.0. For the fluorescence detection, the LNP sample was prepared with fluorescently labeled DSPC-TopFluor (Avanti Polar Lipids, Cat. #810281) substituting 10% DSPC lipid, Cy5 labeled DSPE PEG2000-N-Cy5 (Avanti Polar Lipids, Cat. #810891) substituting 33.3% PEG lipid and 100% siRNA- Cy3 (Sigma-Aldrich, Cat. #SIC003). The LNP samples were formulated by a 3-inlet vortex mixer^35^ at a total flow rate of 1, 10, 20, and 30 mL/min with a lipid to RNA flow rate ratio of 1:3. The final siRNA concentration after LNP formulation was 20μg/mL. The sample was then (at 1 h) dialyzed against 1× phosphate buffered saline (PBS) buffer at pH 7.4 for 12 h under 4°C using dialysis tubes with a molecular weight cutoff (MWCO) of 3,500 Pur-A-Lyzer dialysis kit, Sigma-Aldrich, Cat. #PURD35050). Following dialysis, the LNPs were analyzed by Cylindrical Illumination Confocal Spectroscopy (CICS).^36^ The instrumentation and single-nanoparticle analysis have been developed in our previous work and are described in detail.^15,20^ Briefly, LNP samples were diluted sufficiently to allow for single particles or siRNA molecules to go through the detector one at a time. The 3-color fluorescence signals of each event were used to differentiate LNP populations into siRNA-encapsulated LNPs, empty LNPs, and free unencapsulated siRNAs. By applying the fluorescence deconvolution to the fluorescence distributions of siRNA encapsulated LNPs against a baseline of single siRNA run separately, the siRNA payload distribution on the single LNP level was obtained.

### Molecular dynamics simulations

We modified Cooke and Deserno 3-bead lipid model^37^ by introducing attractive head-to-head interactions to promote nanoparticle formation. Strength and cut-off values for these attractive head-to-head interactions are provided in SI. siRNA was included at a nitrogen-to-phosphate (N/P) ratio of 6, and 1.5% of the lipid was PEGylated, using the same stoichiometry as the experimental setup. The 22 base-pair siRNA and 2000 Da PEG were represented with a bead-spring model. To match experimental conditions, 50% of the lipids carried a positive charge, interacting with the negatively charged siRNA via Debye–Hückel interactions at a salt concentration of 0.025M.^38^ Simulations were conducted using a Langevin thermostat ensuring accurate diffusion behavior of RNA and lipids, detailed are provided in the supporting information (SI). All simulations were performed using LAMMPS,^39^ and OVITO was used for visualization.^40^ RNA–lipid and lipid–lipid contacts (Figure 2B) were counted within a distance 1.7 lj unit, this corresponds to the first valley of lipid-lipid radial distribution functions (see SI). These values were normalized by the coordination number of RNA and lipids in the first solvation shell. Additional simulation setup details and parameters are provided in the SI.

### Kinetic Monte Carlo simulations

We assume that during mixing time $\tau_{\text{M}}$, most lipids form spherical nanoparticles and that after $\tau_{\text{M}}$, the solution is well-mixed. Consequently, the rate per unit concentration at which two spherical nanoparticles with radii $R_{i}$ and $R_{j}$ collide due to diffusion is $K_{\text{collide}}=\frac{2k_{\text{B}}T}{3\eta }\frac{{\left(R_{i}+R_{j}\right)}^{2}}{R_{i}R_{j}},$^41^ with $k_{B}$ is the Boltzmann constant, η the viscosity, and T the absolute temperature. Assuming Arrhenius behavior, the rate at which two specific LNPs in a volume $V_{\text{system}}$ merge is

(1) $$k_{ij}\left(R_{i},R_{j},\Phi_{i},\Phi_{j}\right)=K_{\text{collide}}\left(R_{i},R_{j}\right)e^{-\frac{E_{\text{b}}\left(R_{i},R_{j},\Phi_{i},\Phi_{j}\right)}{k_{B}T}}/V_{\text{system}}.$$

Here $E_{b}$ is the fusion barrier between two LNPs with compositions $\Phi_{i},\Phi_{j}$ (volume fractions of different components in LNPs); $E_{b}$ is modeled by the summation of PEG–PEG repulsion $\left(E_{\text{PEG}}\right)$ and Derjaguin-Landau-Verwey-Overbeek (DLVO) interaction (W),^42^ $E_{\text{b}}=E_{\text{PEG}}+W$. We consider that PEG chains are mobile on the LNP and for the two particles to merge the PEG must be excluded from the interaction area, thus, the free-energy barrier can be calculated as an entropic penalty to exclude PEG chains from the interaction area,

(2) $$E_{\text{PEG}}\left(R_{i},R_{j},\phi_{i}^{\text{PEG}},\phi_{j}^{\text{PEG}}\right)=\frac{2\pi k_{\text{B}}TR_{\text{F}}}{3v_{0}\left(1-f_{w}\right)}R_{i}R_{j}\frac{R_{i}\phi_{i}^{\text{PEG}}+R_{j}\phi_{j}^{\text{PEG}}}{R_{i}+R_{j}}.$$

Here $f_{w}=0.2$ is the water volume fraction in LNPs^15^, $v_{0}$ the volume per lipid, $R_{\text{F}}$ the Flory radius of PEG determined by the PEG size,^32,33^ and $\phi_{i}^{\text{PEG}}$ the PEG fraction in particle i. The DLVO interaction energy is a sum of van der Waals and electrostatic interactions^42^:

(3) $$W\left(d,R_{i},R_{j}\right)=-\frac{AR_{i}R_{j}}{6d\left(R_{i}+R_{j}\right)}+\epsilon \frac{R_{i}R_{j}\left(\psi_{i}^{2}+\psi_{j}^{2}\right)}{4\left(R_{i}+R_{j}\right)}\left[\frac{2\psi_{i}\psi_{j}}{\psi_{i}^{2}+\psi_{j}^{2}}\text{log}\left(\frac{1+e^{-\frac{d}{l_{D}}}}{1-e^{-\frac{d}{l_{D}}}}\right)+\text{log}\left(1-e^{-\frac{d}{l_{D}}}\right)\right],$$

with A is the Hamaker constant, $l_{\text{D}}$ the Debye screening length, ψ the surface electrostatic potential, ϵ the permittivity of solvent, and d the surface-to-surface distance between LNPs. The average time for a fusion event to occur is $\tau =1/\sum k_{ij}$, where the sum proceeds over all LNP pairs in the system. In the kMC implementation, a fusion event is chosen randomly according to the rate $k_{ij}$, with the time increment determined by τ. Each simulation was initialized with 2500 LNPs. This follows our previous work.^15^

### Charge regulation

The potentials ψ in the DLVO equation (Eq. 3) depend on the exact composition and size of the LNP and must be calculated by solving the charge regulation relations^43^. Using the Donnan approximation^44^ we approximate the potential ψ anywhere inside the LNP is a constant and equal to the surface potential $\psi_{0}$. Thus, the charge density is,

(4) $$\rho =\left(1-f_{\text{w}}\right)\left[\alpha_{\text{MC}3}\rho_{\text{MC}3}\phi^{\text{MC}3}+\alpha_{\text{RNA}}\rho_{\text{RNA}}\phi^{\text{RNA}}\right]+2f_{\text{w}}c_{\text{salt}}N_{\text{A}}\text{sinh}\frac{e_{0}\psi_{0}}{k_{\text{B}}T},$$

Here $f_{w}$ is the water volume fraction, $\rho_{\text{MC}3}$ and $\rho_{\text{RNA}}$ are the charge density of cationic lipid and siRNA, $\alpha_{\text{MC}3}$ and $\alpha_{\text{RNA}}$ are the degree of ionizations, $\phi^{\text{MC}3}$ and $\phi^{\text{RNA}}$ are volume fractions, $N_{\text{A}}$ is the Avogadro number, and $e_{0}$ is the elementary charge. When the Debye screening length is $l_{\text{D}}$ for a particular salt concentration, the surface potential depends on the charge density ρ as:

(5) $$\frac{e_{0}\psi_{0}}{k_{B}T}=\frac{4\pi R^{2}\rho l_{\text{D}}}{3\left(1+\frac{R}{l_{\text{D}}}\right)}.$$

A root-finding approach is used to determine the charge density that satisfies the charge regulation equations (Eqs. 4 and 5). To make the model usable for all salt concentration, a surrogate neural network regression model is trained with data varying nanoparticle sizes, RNA fractions, and salt concentrations. The model efficiently predicts surface potential across a broad parameter space. This trained data is provided as a part of the kMC code and predictive toolbox. Details on ML modeling of the charge regulation are provided in the method section

### Feature importance calculations

A random forest regression model was implemented to analyze the influence of formulation parameters on LNP size and the ratio of empty LNPs. The input variables included initial particle size, PEG MW, PEG fraction, and salt concentration, while the output variables were LNP size and the ratio of empty LNPs obtained from kMC simulations. Prior to training, all input variables were standardized. Separate random forest models were trained for each output variable (LNP final size, ratio of empty LNPs and payload distribution variance). The models were evaluated using 5-fold cross-validation with root mean squared error (RMSE) as the performance metric. Feature importance was determined using the mean decrease in impurity (Gini importance) from the trained models. All analyses, including data preprocessing, model training, cross-validation, and visualization, were conducted in Python using the scikit-learn machine learning package.

## Supplementary Material

Supplement 1

The Supporting Information includes detailed experimental procedures, molecular dynamics (MD) simulation parameters, kinetic Monte Carlo (kMC) simulation methodology, and machine learning approaches for charge regulation modeling. It also contains details of the predictive toolbox for LNP size and siRNA payload distribution.

## Data Availability

Research data are available on request.
